# Exosomes and Triple-Negative Breast Cancer: Current Knowledge and Clinical Significance

**DOI:** 10.3390/ijms27041918

**Published:** 2026-02-17

**Authors:** Maria Loukopoulou, Anastasia Kottorou, Angelos Koutras, Foteinos-Ioannis Dimitrakopoulos

**Affiliations:** 1 Molecular Oncology Laboratory, Medical School, University of Patras, 26504 Patras, Greece; maria.m.loukopoulou@gmail.com (M.L.); agathonisiotisa@yahoo.gr (A.K.); 2 Division of Oncology, Department of Medicine, University Hospital of Patras, 26504 Patras, Greece

**Keywords:** exosomes, cancer, TNBC, triple-negative breast cancer, extracellular vesicles

## Abstract

Exosomes, acting as vital mediators of cellular communication and carriers of diverse biomolecular cargo, are increasingly documented as important participants in cancer pathogenesis and progression. When it comes to triple-negative breast cancer (TNBC), a disease that comes with significant therapeutic hurdles, finding new, non-invasive biomarkers is absolutely crucial. This systematic review considers recent research, focusing on the role of exosomal biomarkers in diagnosing, predicting prognosis and foreseeing treatment response in TNBC patients. After an extensive search across PubMed and Google Scholar, we found many exosomal molecules showing great promise for early detection, tracking disease progression and tailoring treatments. This truly highlights liquid biopsy as a valuable, minimally invasive tool. However, there are still some big challenges to treat. These include variations in methodology, the sheer diversity of samples studied and the prevalence of research in specific populations, all of which make it harder to generalize findings. It has been suggested that future research must prioritize protocol standardization, achieving a deeper understanding of underlying biological mechanisms and, crucially, developing combinatorial biomarker panels. Ultimately, the successful translation of exosomal biomarkers into clinical practice will significantly advance personalized medicine in TNBC, leading to improved patient outcomes and an enhanced quality of life.

## 1. Exosomes: Silent Messengers of Cellular Communication

At the heart of cellular communication and complex pathological interplays, exosomes seem to take on the role of tiny messengers capable of revolutionizing our understanding of intercellular dialogs [1]. Exosomes are nanovesicles that possess a spherical morphology, ranging between 30 and 150 nanometers, encased within a bilayer phospholipid membrane similar to typical cellular membranes [2].

Exosomes have been isolated from all body fluids (e.g., blood, plasma, cerebrospinal fluid, breast milk and even urine), making them easily accessible for clinical study [3]. In exosome research, plasma and serum are the most commonly used sources of blood-derived samples. The choice between them can have a significant impact on exosome yield, purity and the profile of molecular biomarkers [4]. Plasma is generally preferred because clotting during serum preparation activates platelets, leading to the release of additional extracellular vesicles that may contaminate the exosome fraction [5]. These differences can affect both the reproducibility and interpretation of results, emphasizing the importance of careful sample selection and standardized collection protocols, particularly when aiming for clinical applications [6].

These nanovesicles are secreted by cells dynamically and continuously under normal physiological conditions as well as when the state of a cell is compromised. This means they are uniquely capable of mirroring the state of the parent cell [7]. Through these vesicles, valuable information travels to distant cells and tissues, contributing to the organism’s homeostasis. However, when this equilibrium is disturbed, as in cancer, they can also foster pathological processes [8].

**Figure 1:**
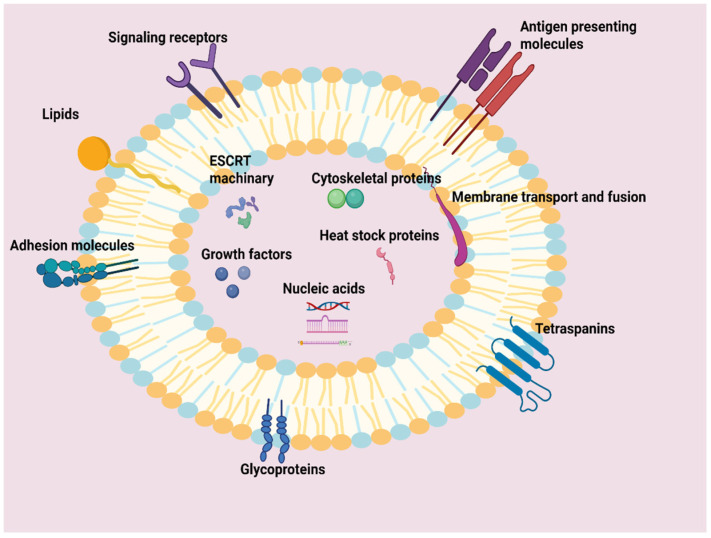
An exosome, enveloped by a phospholipid bilayer, hosts a rich and diverse molecular cargo. This includes membrane proteins (such as tetraspanins, antigen-presenting molecules, adhesion molecules, glycoproteins, signaling receptors and membrane transport/fusion proteins), internal proteins (such as heat shock proteins, cytoskeletal proteins, ESCRT system components and growth factors), as well as various types of nucleic acids and lipids. This composition reflects the cellular origin and the exosome’s role in intercellular communication (Created with BioRender.com).

Exosomes invariably contain a remarkably complex and diverse “cargo” depending on their functional versatility (Figure 1). The categories of this cargo are mainly proteins, nucleic acids and lipids, each responsible for a specific function or set of functions [9].

The proteins inside exosomes exhibit considerable diversity. Some proteins are universal in all exosomes and function as their “identity markers,” such as tetraspanins CD9, CD63 and CD81, RAB GTPases, Alix and HSP60/90 (heat shock proteins) [10,11]. Other proteins are more specific and indicate the type of cell comprising them, such as MHC (major histocompatibility complex) Class I and II, responsible for antigen presentation [12]. Exosomes can also carry membrane-bound signaling receptors like FasL (Fas ligand) and TNFR (tumor necrosis factor receptor), which participate in cell death pathways. Other molecules include adhesion molecules like integrins, which mediate attachment to the extracellular matrix and influence cell adhesion and migration. The diverse transport and communication functions of exosomes are the result of the cooperation of glycoproteins involved in cell communication and recognition, of cytoskeletal proteins belonging to the ESCRT (Endosomal Sorting Complexes Required for Transport) system, of membrane transport and fusion proteins, of growth factors and cytokines [13,14].

Regarding nucleic acids, exosomes act as containers storing genetic information. Exosomes can carry many types of RNA, including mRNA, microRNA (miRNA), lncRNA (long non-coding RNAs), tRNA, snRNA (small nuclear RNA), snoRNA (small nucleolar RNA) and circRNA (circular RNAs) [15]. Among these, miRNAs are the most abundant in number and are essential in the regulation of gene expression that affects processes such as hematopoiesis, angiogenesis and exocytosis. The mRNAs conveyed by them are coding for protein synthesis, making exosomes a pronounced means of transferring genetic instructions to distant cells that may contribute to protein replenishment, cell differentiation, or microenvironment modification [16,17]. Moreover, exosomes also carry PIWI-interacting RNAs (piRNAs). These non-coding RNAs play a vital role in safeguarding the genome from transposable elements, thereby ensuring genetic stability and notably influencing tumor development [18,19].

Finally, the exosomal membrane bilayer is exceptionally rich in lipids, including sphingomyelin, cholesterol and ceramides. These are not just structural components; they are crucial for maintaining the exosomal structure’s stability, facilitating their secretion and ensuring the precise sorting of their internal cargo. Furthermore, they actively contribute to intercellular signaling [20].

## 2. Exosome Biogenesis: A Complex Cellular Process

The production of exosomes is a strictly regulated and dynamic process, guided by specific molecular pathways. Understanding the mechanisms of exosome biogenesis and their composition is of vital importance, as it illuminates their functional activities and paves the way for future clinical applications [21,22].

**Figure 2:**
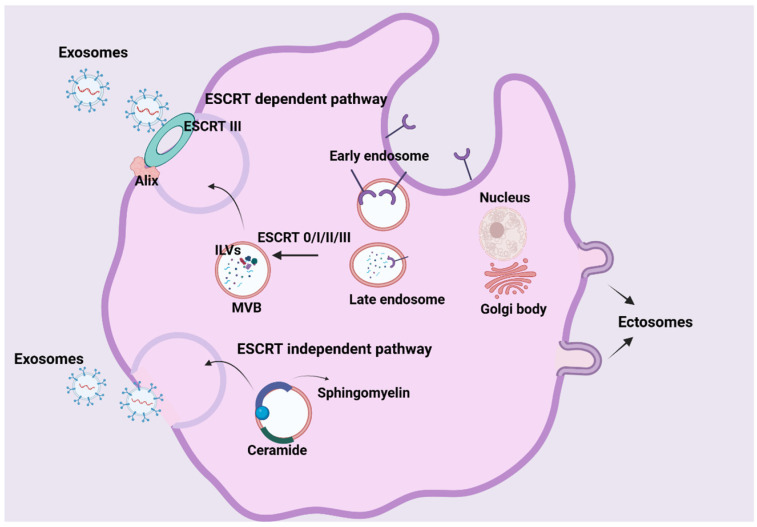
Exosome biogenesis begins with the formation of early endosomes and continues with their maturation into late endosomes and MVBs. Within MVBs, ILVs are formed via both ESCRT-dependent and ESCRT-independent pathways. The process culminates with the fusion of MVBs with the plasma membrane and the release of exosomes into the extracellular space (created with BioRender.com).

Exosome formation begins with endocytosis at the plasma membrane’s surface, leading to the creation of early endosomes (Figure 2) [23,24]. These early endosomes function as sorting stations for internalized cargo, maturing and transforming into late endosomes. At this stage, they form multiple inward membrane invaginations that encapsulate specific proteins, nucleic acids and other substances, thereby generating intraluminal vesicles (ILVs), the precursors of exosomes. Subsequently, late endosomes containing multiple ILVs develop into multivesicular bodies (MVBs) [25,26].

The fate of MVBs varies. While some MVBs fuse with lysosomes for the degradation of their contents, others are destined for the secretion of ILVs into the extracellular space (Figure 2) [27]. These released ILVs are then termed exosomes. The secretion of MVBs from the cell involves three key steps: targeted transport of MVBs, tethering of MVBs to the plasma membrane, and fusion of the MVB’s limiting membrane with the plasma membrane (Figure 2) [25]. The proper functioning of this entire process relies on the surface proteins of the MVB [28].

The biogenesis of ILVs and MVBs is primarily guided by two main and distinct, yet sometimes overlapping, mechanisms: the ESCRT-dependent and the ESCRT-independent pathways (Figure 2) [29].

- ESCRT-dependent pathway: This intricate pathway is precisely orchestrated by a large multiprotein complex comprising around thirty different types of proteins. The core function of the ESCRT machinery is to selectively sort specific components into ILVs. The ESCRT system itself is made up of four main complexes—ESCRT-0, ESCRT-I, ESCRT-II and ESCRT-III—each playing a unique and vital role [30].

ESCRT-0: It is responsible for cargo aggregation in a ubiquitination-dependent process, recognizing ubiquitinated cargo proteins. It contains the HRS (Hepatocyte Growth Factor-Regulated Tyrosine Kinase Substrate) protein, which can interact with STAM.

ESCRT-I and ESCRT-II: They function synergistically to induce the encapsulation of specific molecules from the endosomal membrane through budding. HRS recruits TSG101 to ESCRT-I and then ESCRT-I recruits ESCRT-III via ESCRT-II or ALIX.

ESCRT-III: It drives vesicle scission and forms a filamentous structure that promotes the inward budding and fission of ILVs from the endosomal membrane. The ESCRT-III complex is disassembled by the VPS4 complex and is subsequently recycled.

ESCRT-associated proteins: Proteins such as ALIX and VPS4 also contribute to MVB biogenesis by regulating cargo sorting, membrane remodeling and ESCRT disassembly. The ALIX protein can interact with syndecan-syntenin and participate in regulating exosome formation [10,30,31].

b. ESCRT-independent pathway: In addition to the ESCRT-dependent pathway, ILVs and MVBs can also be generated by ESCRT-independent mechanisms (Figure 2). These mechanisms involve lipids, such as sphingomyelin, cholesterol and ceramides, as well as tetraspanins and HSPs [26]. Exosomes are rich in these lipids, whose composition resembles that of membrane lipid rafts. Evidence suggests that lipid raft components play fundamental roles in ESCRT-independent ILV formation [26,32,33].

It is important to note that the ESCRT-dependent and ESCRT-independent pathways are not mutually exclusive. They can even operate within the same MVB, leading to the production of distinct ILV populations enriched with different cargo. This suggests that the pathways may intersect to some extent, which can depend on the cell type, its environment and its cargo [34].

## 3. Exosomes: Important Contributors in Triple-Negative Breast Cancer Pathogenesis

Triple-Negative Breast Cancer (TNBC) is a particularly aggressive and heterogeneous subtype of breast cancer. It is characterized by the absence of estrogen receptor (ER), progesterone receptor (PR) and HER2 expression, which drastically limits available targeted therapies [35]. This clinical challenge makes it imperative to delve deeper into the underlying molecular mechanisms of the disease, enabling the development of new, more effective therapeutic strategies [36].

In light of this imperative need, exosomes have emerged as important factors in the pathobiology of many tumor types [37,38,39], and recently, of TNBC. Through their ability to transfer a wide range of biomolecules and influence cell-to-cell signaling, they actively participate in shaping the tumor microenvironment (TME) and regulating the behavior of cancer cells at every stage of the disease [40,41].

Specifically, exosomes, through proteins and non-coding RNAs, actively contribute to tumor growth by enhancing cancer cell proliferation, inflammation and angiogenesis [42]. Exosomes play a significant role in TNBC metastasis, encompassing both local invasion and the establishment of distant metastatic niches. Exosomes promote epithelial–mesenchymal transition (EMT), a critical step for cancer cell invasion and dissemination [43,44]. Furthermore, by transferring biomolecules from their cells of origin, they can target specific remote tissues and create a “permissive” pre-metastatic environment, thereby facilitating the engraftment and growth of metastatic cells. A characteristic example is the involvement of exosomal integrins in predicting and shaping these metastatic sites [45,46].

Moreover, exosomes are a significant factor in the development of drug resistance, a major obstacle to effective TNBC treatment [47]. Although chemotherapy remains the primary therapeutic approach, a significant percentage of patients (approximately 70%) do not achieve a complete response and in some cases, chemotherapy-induced metastasis is even observed [48]. The development of chemoresistance by cancer cells is a complex process involving multiple mechanisms [49]. In this context, exosomes emerge as critical factors, as they actively participate in intercellular communication within the tumor microenvironment [50]. Experimental evidence shows that exosomes derived from chemoresistant breast cancer cells can transfer resistance-associated molecular cargo, including miRNAs and lncRNAs, to drug-sensitive cells, thereby modulating drug response pathways and promoting chemoresistant phenotypes in recipient cells [51,52]. Specifically, three main exosomal mechanisms contribute to increased chemotherapeutic drug efflux: the direct efflux of drugs from cells, the transfer of membrane-embedded drug efflux pumps and the transfer of drug-metabolizing enzymes, leading to their deactivation [53].

## 4. Exosomal Biomarkers in ΤΝBC

TNBC, as an aggressive subtype of breast cancer with limited therapeutic options, highlights the urgent need for reliable non-invasive biomarkers [54]. In this context, exosomes represent one promising approach among several emerging tools. Due to their ability to carry a dynamic cargo of biomolecules that reflects the molecular state of the tumor, they have been investigated as potential tools to support early diagnosis, provide prognostic information on disease progression and help predict treatment response, contributing decisively to personalized medicine in TNBC [55].

### 4.1. Exosomes as Diagnostic Biomarkers in TNBC

Early and non-invasive detection of TNBC is crucial for improving patient outcomes. Exosomes offer a unique molecular mirror of the tumor and thus, various studies have identified diverse exosomal biomolecules with diagnostic value in TNBC [56,57].

### 4.2. Exosomal microRNAs (miRNAs) as Diagnostic Biomarkers in TNBC

Exosomal miRNAs are key regulators of gene expression transported via exosomes, making them ideal candidates for non-invasive biomarkers [58]. Numerous studies have investigated their diagnostic value in TΝBC (Table 1a). Diverse exosomal miRNAs with a potential diagnostic role have been identified in breast cancer patients, irrespective of subtype. Specifically, the gastrointestinal system-derived miRNA (miGISig) and miR-122-5p were observed to be elevated in the plasma exosomes of patients, demonstrating effective diagnostic accuracy [59,60]. Similarly, increased levels of exosomal miR-3662, miR-146a, miR-1290, miR-200c and miR-372 were found in the serum of BC patients [61,62,63]. Exosomal miR-92b-5p in serum was observed to be elevated in BC patients compared with healthy controls and was correlated with disease stage [64], while exosomal miR-148a in serum was found to be significantly reduced in BC patients [65].

Of particular interest is miR-21, which is strongly recommended for breast cancer screening, and its diagnostic value in breast cancer has been confirmed by meta-analyses, suggesting improved accuracy through its combination with other miRNAs [66]. Indeed, miR-1246 and miR-21 were detected at significantly higher levels in the plasma exosomes of patients, with their combination serving as a superior diagnostic marker compared to their individual levels [67]. However, it is noteworthy that another study found the expression of miR-21 in urinary exosomes to be significantly lower in breast cancer patients than in controls [68]. Although both studies employed qRT-PCR for miR-21 quantification, the discrepancy in results likely arises from the different exosome sources (plasma vs. urine), underscoring the impact of sample type on exosomal biomarker levels.

Additionally, diagnostic accuracy is enhanced by the use of biomarker panels. One such panel, consisting of four urinary microRNAs (miR-424, miR-423, miR-660 and let7-i), was identified as a highly specific and sensitive tool for distinguishing breast cancer patients from healthy controls [69]. Furthermore, a panel of four exosomal miRNAs (miR-9, miR-16, miR-21 and miR-429) was found to be significantly elevated in early-stage breast cancer patients, irrespective of subtype, compared to healthy controls [70].

Beyond these general findings in breast cancer, numerous exosomal miRNAs have been specifically identified for their diagnostic role in TNBC. For instance, serum levels of sEV-miR-373 have been found significantly elevated in TNBC patients compared to other breast cancer subtypes [61]. Similarly, significantly higher levels of plasma exosomal miR-376c and miR-382 have been observed in TNBC patients relative to healthy controls [71]. A comprehensive study identified six upregulated miRNAs (hsa-miR-148a-5p, hsa-miR-200a-5p, hsa-miR-210a-3p, hsa-miR-378a-3p, hsa-miR-483-5p and hsa-miR-7110-5p) and two downregulated miRNAs (hsa-miR-92b-3p and hsa-miR-150-5p) in the plasma exosomes of TNBC patients versus healthy controls [72]. Additionally, the expression of miR-335-5p in the serum exosomes of TNBC patients has been found to be lower, while FLT1 was higher than in the control group [73]. Notably, miR-150-5p accurately distinguished Luminal A and TNBC subtypes [74]. Additionally, two exosomal oncomiRs, hsa-miR-1180 and hsa-miR-4728, were found to be significantly elevated in tumor tissue samples from TNBC patients [75]. Finally, a panel of six miRNAs (miR-21, miR-221, miR-210, miR-195, miR-145 and Let-7a) was proposed as a minimally invasive biomarker for the early detection of TNBC, with miR-21, miR-221 and miR-210 showing significant overexpression and miR-195 and miR-145 being downregulated, all correlating well with various clinicopathological and demographic factors [76].

### 4.3. Exosomal circRNAs and lncRNAs in Diagnosis of TNBC

CircRNAs and exosomal lncRNAs can also offer valuable information for the early diagnosis and monitoring of TNBC (Table 1b,c). The expression of circHSDL2 was significantly elevated in serum exosomes and tumor tissues of TNBC patients compared to healthy controls [77]. Concurrently, circSIPA1L3 has been recognized with high diagnostic value, showing significantly increased levels in serum exosomes of breast cancer patients more generally [78]. Correspondingly, the expression level of circSTIL in patients’ plasma exosomes showed potential diagnostic value in distinguishing TNBC from non-TNBC subtypes [79]. Furthermore, higher expression of circPSMA1 was observed in serum exosome samples from TNBC patients, as well as in TNBC cell lines, compared to non-TNBC patients and cell lines [80].

Regarding lncRNAs, lncRNA XIST in serum has emerged as a strong diagnostic indicator. Serum exo-XIST levels were capable of distinguishing TNBC patients from the healthy control group. Interestingly, these levels significantly decreased after the excision of primary breast tumors, indicating their association with tumor presence [81]. Additionally, the expression level of lncRNA SUMO1P3 in serum exosomes was significantly higher in TNBC patients compared to non-TNBC patients, patients with benign breast disease and healthy controls [82].

### 4.4. Exosomal Proteins as Diagnostic Biomarkers in TNBC

Exosomal protein cargo represents another promising category of biomarkers for TNBC diagnosis and several studies have highlighted the diagnostic value of some of them (Table 1d). Expression levels of the exosome tetraspanin CD151 in a serum derived from TNBC were significantly higher than those from healthy individuals [83]. Furthermore, serum exo-AnxA2 levels could serve as a suitable diagnostic tool for aggressive breast cancer, especially in TNBC patients; the concentration of exo-AnxA2 was also significantly increased in serum samples from breast cancer patients compared to healthy individuals [84]. Lastly, three exosomal membrane/surface proteins—glucose transporter 1 (GLUT-1), glypican 1 (GPC-1) and ADAM metallopeptidase domain 10 (ADAM10)—were recognized as potential breast cancer biomarkers in a study conducted on cell lines [85].

**Table 1:** (**a**) Exosomal miRNAs for diagnosis in TNBC. (**b**) Exosomal lncRNAs for diagnosis in TNBC. (**c**) Exosomal circRNAs for diagnosis in TNBC. (**d**) Exosomal proteins for diagnosis in TNBC.

**(a)**				
**Biomarker**	**Sample Type**	**N (Patients)/N (Controls/Others)**	**Correlation**	**Reference**
miGISig	Plasma	20 BC/10 HC	Elevated in plasma exosomes of Chinese BC patients	[59]
miR-122-5p	Plasma	257 BC/257 HC	Elevated in plasma exosomes of Chinese BC patients (38.9% TNBC)	[60]
miR-3662	Serum	60 BC/20 HC	Increased levels in the serum of Chinese BC patients	[63]
miR-146a				
miR-1290				
miR-200c	Serum	98 BC/45 benign/79 HC	Increased levels in the serum of Chinese BC patients	[62]
miR-372	Serum	50 Invasive BC/12 HC	Increased levels in serum of German BC patients (21% TNBC)	[61]
miR-92b-5p	Serum	59 BC/53 HC	Elevated in Taiwanese BC patients vs. HC; correlated with disease stage	[64]
miR-148a	Serum	125 BC/50 benign/40 HC	Significantly reduced in Chinese BC patients	[65]
miR-21 miR-1246	Plasma	16 BC/16 HC	Significantly higher in plasma exosomes of American BC patients	[67]
miR-21	Urine	22 BC/26 HC	Significantly lower in Japanese BC patients than in controls	[68]
miR-424	Urine	69 BC/40 HC	Highly specific/sensitive tool for distinguishing BC from HC in German patients (16 TNBC/69)	[69]
miR-423				
miR-660				
let-7i				
miR-9	Plasma	62 BC/20 HC	Significantly elevated in early-stage South Korean BC patients (15/62)	[70]
miR-16				
miR-21				
miR-429				
miR-373	Serum	168 primary BC/19 benign/28 HC	Significantly elevated in German TNBC patients vs. other BC subtypes	[61]
miR-376c	Serum	224 TNBC/20 HC	Elevated in German TNBC patients relative to healthy controls	[71]
miR-382				
hsa-miR-148a-5p	Plasma	27 BC/3 HC	Upregulated in Chinese TNBC patients vs. HC	[72]
hsa-miR-200a-5p				
hsa-miR-210a-3p				
hsa-miR-378a-3p				
hsa-miR-483-5p				
hsa-miR-7110-5p				
hsa-miR-92b-3p			Downregulated in Chinese TNBC patients vs. HC	
hsa-miR-150-5p				
miR-335-5p	Serum	56 TNBC/HC not specified	Lower expression in Chinese TNBC patients than in HC	[73]
miR-150-5p	Serum	31 BC (16 LA. 15 TNBC)/16 HC	Accurately distinguished LA and TNBC subtypes in Brazilians	[74]
hsa-miR-1180	Tumor Tissue	15 TNBC tumor/15 adjacent normal	Significantly elevated in Indian TNBC tumor tissue	[75]
hsa-miR-4728				
miR-21 miR-221 miR-210	Serum	85 TNBC/85 HC	Overexpressed in Indians TNBC patients; early detection biomarker	[76]
	Tissue	85 TNBC/85 adjacent normal		
miR-195 miR-145 Let-7a	Serum	85 TNBC/85 HC	Downregulated in Indians TNBC patients; early detection biomarker	
	Tissue	85 TNBC/85 adjacent normal	Overexpressed in Indians TNBC patients; early detection biomarker	
**(b)**				
**Biomarker**	**Sample Type**	**N (Patients)**/**N (Controls/Others)**	**Correlation**	**Reference**
lncRNA XIST	Serum	91 TNBC/50 HC	Distinguishes TNBC patients from HC in Chinese; decreased after tumor excision	[81]
SUMO1P3	Serum	130 TNBC/60 non-TNBC/60 benign/50 HC	Higher in Chinese TNBC patients compared to non-TNBC, benign and HC	[82]
**(c)**				
**Biomarker**	**Sample Type**	**N (Patients)**/**N** **(Controls/Others)**	**Correlation/Finding**	**Reference**
circHSDL2	Serum Exosomes and Tumor Tissue	43 TNBC (serum)/Not specified HC (serum)/20 TNBC tumor	Significantly elevated in serum exosomes and tumor tissues of TNBC patients vs. HC	[80]
circSIPA1L3	Serum	50 BC/50 HC	Significantly increased levels in the serum exosomes of Chinese BC patients	[78]
circSTIL	Plasma	59 TNBC/40 non-TNBC	Diagnostic value in distinguishing TNBC from non-TNBC subtypes in Chinese	[79]
circPSMA1	Serum and Cell Lines	20 TNBC/20 non-TNBC	Increased expression in Chinese TNBC patients and TNBC cell lines vs. Non-TNBC patients/lines	[77]
**(d)**				
**Biomarker**	**Sample Type**	**N (Patients)**/**N** **(Controls/Others)**	**Correlation**	**Reference**
CD151	Serum	10 TNBC/17 HC	Higher expression in Chinese TNBC patients than in HC	[83]
exo-AnxA2	Serum	169 BC/68 HC	Concentration significantly increased in serum samples from American BC patients vs. HC	[84]
FLT1	Serum	56 TNBC/Not specified HC	Higher expression in Chinese TNBC patients than in HC	[73]
GLUT-1	BC Cell Line and non-tumorigenic breast cell line		Increased expression in BC cell lines	[85]
GPC-1				
ADAM10				

Abbreviations: BC: breast cancer, HC: healthy control, TNBC: triple-negative breast cancer.

## 5. Exosomes as Prognostic Indicators in TNBC

Beyond their diagnostic role, exosomes are emerging as reliable prognostic indicators in TNBC. Their study allows for the prediction of recurrence, disease progression, the occurrence of metastases and estimates patients’ overall survival (OS) and disease-free survival (DFS).

### 5.1. Exosomal miRNAs as Prognostic Indicators in TNBC

Decreased expression of exosomal miR-148a in serum and exosomal miGISig in plasma has been closely linked to an unfavorable clinical outcome in breast cancer patients (Table 2a) [59,65]. Conversely, high expression of a panel of four miRNAs—miR-4448, miR-2392, miR-2467 and miR-4800—in serum was associated with longer OS (overall survival) in TNBC patients [86]. Εxosomal miRNA-21 and miR-105 expression levels in serum were higher in metastatic BC patients compared to non-metastatic patients and healthy controls [87].

Furthermore, the expression of miR-335-5p in serum exosomes was related to histological grade, differentiation degree and lymph node metastasis. Specifically, low expression of miR-335-5p was associated with significantly shorter OS in TNBC patients, while high expression of its target gene, FLT1, was linked to a lower survival rate [73]. Additionally, high expression of Akt1 combined with low expression of mir-637 in serum exosomes was strongly correlated with poor prognosis in TNBC patients with lymph node metastasis [80]. Finally, miR-939 in tissue exosomes emerged as a prognostic factor for DFS, particularly in combination with lymph node involvement (N), multifocal occurrence and tumor size, suggesting a synergistic risk of recurrence in patients with high miR-939 expression and lymph node involvement [88].

### 5.2. Exosomal lncRNAs and circRNAs as Prognostic Indicators in TNBC

Several studies have associated the increased expression of specific circRNAs with an unfavorable prognosis and more aggressive TNBC behavior (Table 2c). Specifically, the increased expression of exosomal circSIPA1L3 in serum was associated with shorter OS in BC patients [78]. Similarly, high expression levels of exosomal circSTIL in plasma were correlated with worse DFS in TNBC patients, although no association with OS was found [89]. Furthermore, exosomal circHSDL2 in the serum of TNBC patients appeared to promote proliferation, infiltration and metastasis in TNBC cells, while the increased expression of exosomal circMIB1, derived from CAFs (cancer-associated fibroblasts), was significantly associated with shorter OS and DFS in TNBC patients [77,90].

Another significant prognostic biomarker is the exosomal lncRNA XIST, whose expression levels have been shown to be significantly higher in patients with recurrent TNBC compared to those with non-recurrent TNBC (Table 2b). High expression of exo-XIST in serum was associated with worse OS, while its expression significantly decreased after primary tumor excision and increased after recurrence, indicating that exo-XIST can serve as a non-invasive biomarker for predicting the progression of TNBC patients [81].

Additionally, lncRNA SUMO1P3 in serum exosomes was closely associated with vascular/lymphatic invasion, lymph node metastasis and histological grade. Patients with high levels of lncRNA SUMO1P3 in serum exosomes had worse OS compared to those with low levels [82]. Similarly, exosomal lncRNA LINC00989 in serum was significantly elevated in patients with metastatic TNBC [91]. Higher levels of this biomarker, often in combination with clinical markers such as CEA and CA125, were significantly associated with shorter OS and PFS, establishing it as an independent prognostic factor [91]. Finally, LINC00899 was found to be reduced in plasma exosomes and breast cancer cell lines, correlating with the Ki-67 index, tumor size and the presence or absence of lymph node metastasis [89].

### 5.3. Exosomal Proteins as Prognostic Indicators in TNBC

Additionally, the level of exosomal LDHC (Lactate Dehydrogenase C) in the serum of preoperative breast cancer patients was significantly higher than in postoperative patients (Table 2d). Furthermore, in patients with recurrent disease, the level of exosomal LDHC was elevated, suggesting that exosomal LDHC in serum could be a promising indicator for effective monitoring and prediction of breast cancer recurrence [92]. The exosomal protein AnxA2 (exo-AnxA2) has shown significant prognostic value. Exo-AnxA2 levels were significantly higher in serum samples from TNBC patients with grade III compared to grade II patients. Breast cancer patients with high exo-AnxA2 levels in serum had significantly shorter OS and worse DFS. Moreover, the association of exo-AnxA2 with African American TNBC patients suggests its potential role as a factor contributing to TNBC aggressiveness in these women [84]. Finally, one study showed that high levels of the exosomal proteins EV_APRIL, EV_CXCL13 and EV_VEGF-A in the serum of TNBC patients were associated with significantly shorter OS. Multi-parametric analysis even identified the high level of EV_CXCL13 as an independent prognostic factor for poor OS [93].

**Table 2:** (**a**) Exosomal miRNAs for prognosis in TNBC. (**b**) Exosomal lncRNAs for prognosis in TNBC. (**c**) Exosomal circRNAs as prognostic Biomarkers in TNBC. (**d**) Exosomal proteins as prognostic Biomarkers in TNBC.

**(a)**				
**Biomarker**	**Sample Type**	**N (Patients)/N (Controls/Others)**	**Correlation**	**Reference**
miR-148a	Serum	125 BC and 50 benign/40 HC	Lower expression in Chinese BC patients linked to worse OS and DFS	[65]
miGISig	Plasma	210	Higher expression in Chinese BC patients linked to worse OS	[59]
miR-4448	Serum	24 TNBC	High expression associated with longer OS in Japanese TNBC patients	[86]
miR-2392				
miR-2467				
miR-4800				
miR-21	serum	53 BC	Higher expression in metastatic Spanish BC patients	[87]
miR-105				
miR-335-5p	Serum	56 TNBC	Low expression associated with worse OS in Chinese TNBC patients	[73]
miR-637	Serum	20 TNBC	Low expression (combined with high Akt1) correlated with poor prognosis in Chinese TNBC patients with lymph node metastasis	[80]
miR-939	Tissue	63 TNBC	High expression associated with recurrence risk in Italian breast cancer patients	[88]
**(b)**				
**Biomarker**	**Sample Type**	**N (Patients)/N (Controls/Others)**	**Correlation**	**Reference**
XIST	Serum	91 TNBC	High expression associated with worse OS in Chinese TNBC patients	[81]
SUMO1P3	Serum	130 TNBC	High expression associated with worse OS in Chinese TNBC patients	[82]
LINC00989	Serum	135 TNBC	Elevated in metastatic TNBC; combination with CEA, CA125 associated with shorter OS and PFS in Chinese TNBC patients	[91]
LINC00899	Plasma	119 BC	Correlated with Ki-67 index, tumor size and presence/absence of lymph node metastasis	[89]
**(c)**				
**Biomarker**	**Sample Type**	**N (Patients)/N (Controls/Others)**	**Correlation**	**Reference**
circSIPA1L3	serum	238 BC	Increased levels associated with shorter OS in Chinese BC patients.	[78]
circSTIL	plasma	49 TNBC	High expression associated with worse DFS in Chinese TNBC patients.	[79]
circHSDL2	serum	43 TNBC	Promotes proliferation, infiltration and metastasis in TNBC cells.	[77]
circMIB1	exosomes from CAFs	113 TNBC	Increased expression associated with shorter OS and DFS in Chinese TNBC patients.	[90]
**(d)**				
**Biomarker**	**Sample Type**	**N (Patients)/N (Controls/Others)**	**Correlation**	**Reference**
FLT1	serum	56 TNBC	High expression associated with worse OS in Chinese TNBC patients.	[73]
Akt1	serum	20 TNBC	High expression of Akt1 combined with low expression of miR-637 correlated with poor prognosis in Chinese TNBC patients with lymph node metastasis.	[80]
LDHC	serum	75 BC	Higher levels in Chinese preoperative breast cancer patients than in postoperative patients.	[92]
AnxA2	serum	169 TNBC	Higher levels associated with shorter OS and worse DFS in Americans and higher levels in TNBC patients with Grade III compared to Grade II.	[84]
EV_APRIL	serum	190 TNBC	Higher levels in Korean TNBC patients associated with shorter OS.	[93]
EV_CXCL13				
EV_VEGF-A				

Abbreviations: BC: breast cancer, HC: healthy control, TNBC: triple-negative breast cancer, DFS: disease-free survival, OS: overall survival, CAF: cancer-associated fibroblasts.

## 6. Exosomal Biomarkers as Predictive Indicators in TNBC

Beyond their diagnostic and prognostic roles, exosomes and their biomolecular content may also serve as predictive biomarkers in TNBC. Their ability to dynamically reflect tumor response to various therapeutic approaches, such as chemotherapy and immunotherapy, offers an invaluable tool for treatment individualization. By studying exosomes, researchers aim to identify patients more likely to respond to specific therapies, enable early recognition of treatment resistance and monitor minimal residual disease, ultimately leading to more effective and targeted interventions [94].

### 6.1. Overall Exosome Concentration and Treatment Response

The overall concentration of extracellular vesicles (EVs) in plasma, among which exosomes are particularly critical, has emerged as a significant predictive indicator for the response of BC patients to neoadjuvant chemotherapy (NACT). More specifically, one study showed that EV concentration was 40 times higher in BC patients compared to healthy controls, and that an increase in its concentration after NACT was associated with treatment failure, reduced three-year DFS, and OS. Consequently, this study suggests that plasma EV concentration in BC patients may reflect the status of minimal residual disease (MRD), as well as treatment response [95].

### 6.2. Exosomal RNA as Predictive Indicators for Chemotherapy Response

At the level of **lncRNAs**, SUMO1P3 levels in serum exosomes significantly decreased in cases of chemosensitivity, while remaining unchanged in cases of chemoresistance in TNBC patients (Table 3a) [82]. Correspondingly, LINC00899 in plasma exosomes demonstrated high predictive value for pathological outcome in TNBC patients receiving neoadjuvant treatment, indicating its potential to predict chemotherapy efficacy [89]. Similarly, the expression of circSTIL from plasma exosomes significantly increased in TNBC patients without NAC compared to patients who received NAC [79].

Regarding **miRNAs**, several have emerged as predictive indicators for response to neoadjuvant chemotherapy (NAC). Specifically, the expression of miR-4448 and miR-2392 in serum was significantly higher in patients with pathological complete response (pCR) compared to those with non-pCR. A combined model of these two miRNAs, along with miR-2467-3p and miR-4800-3p, is reported to accurately distinguish between pCR and non-pCR patients in TNBC [86]. Likewise, the upregulation of plasma miR-127 was associated with pCR in BC. While after the first dose of NACT, high expression of miR-141 in plasma was associated with pCR, miR-34a, exo-miR182 and exo-miR-183 predicted non-pCR [96].

Furthermore, the plasma exosomal expression levels of miR-185, miR-4283, miR-5008 and miR-3613 were found to be lower in TNBC patients with no NACT response, whereas miR-1302, miR-4715 and miR-3144 were higher [97]. MiR-770 was highly expressed in chemosensitive tissues and predicted a better prognosis for TNBC, indicating its significant role in regulating chemoresistance and disease progression [98]. Additionally, hsa-miR-6831-5p in serum showed a significant difference in expression between responding and drug-resistant patients to AC-sequential T2 chemotherapy, indicating its potential as a chemosensitivity marker [99]. Finally, during neoadjuvant therapy, miRNA-21 levels in the serum of BC patients were directly correlated with tumor size and inversely with Ki67 expression [87].

### 6.3. Exosomal Proteins as Predictive Indicators for Chemotherapy Response

Regarding exosomal proteins, exosomal ANXA6 levels at baseline were lower in highly sensitive TNBC patients than in resistant TNBC patients when they received gemcitabine-based first-line chemotherapy (Table 3b). This suggests that serum exosomal ANXA6 levels in TNBC patients could be predictive of the response to gemcitabine-based chemotherapy [100].

### 6.4. Exosomal Biomarkers as Predictive Indicators for Immunotherapy Response

Immunotherapy is an emerging therapeutic approach in TNBC and exosomes may be able to predict response to it. Specifically, exosomal PD-L1 has emerged as a significant predictive biomarker (Table 3c). Raimondi et al. have reported that patients treated with atezolizumab plus nab-paclitaxel have a significantly higher number of exosomal PD-L1 mRNA copies per mL at baseline in the case of complete or partial response compared to those with stable or progressive disease [101]. This finding aligns with another study, which demonstrated that plasma exosomes enriched with PD-L1 could serve as a favorable and predictive biomarker for immune checkpoint blockade (ICB) therapies in metastatic TNBC [102].

However, the first study adds a dynamic dimension, showing that an increase in PD-L1 mRNA copies per mL after treatment was associated with significantly shorter progression-free survival and OS. This suggests that while high initial expression may be favorable for response, an increase during or after treatment might indicate the development of resistance or more aggressive disease [101]. Consequently, exosomal PD-L1 seems to be associated with treatment outcome and response to immunotherapy.

The current standard for guiding immunotherapy decisions in TNBC is tissue PD-L1 expression, which, however, has inherent limitations: it reflects expression only at the site and time of the biopsy and may not capture the spatial heterogeneity of tumors [103,104]. In contrast, exosomal PD-L1 in plasma offers a non-invasive, dynamic alternative. It allows for repeated sampling, reflects PD-L1 from multiple tumor sites, and may provide earlier insights into treatment response or the emergence of resistance [105,106]. By complementing tissue-based analyses, exosomal PD-L1 has the potential to enhance predictive accuracy for immunotherapy, although further studies are needed to determine optimal measurement methods and validate its utility across diverse patient populations [107].

## 7. Conclusions and Future Perspectives

Τhis review highlights the multifaceted role of exosomes as promising biomarkers in TNBC. Due to the heterogeneity, aggressive nature and lack of targeted therapies for this particular disease, the need for novel, non-invasive biomarkers is escalating [108]. Diverse RNA molecules (such as miRNAs, lncRNAs, circRNAs) as well as proteins within exosomes have been studied for their role in the early diagnosis of TNBC, the prediction of disease progression and the response to therapies. In addition, exosomes offer a minimally invasive and dynamic method for disease monitoring [109]. However, the integration of exosomal biomarkers into clinical practice faces significant challenges and limitations. Initially, a notable heterogeneity has been observed across studies, both regarding exosome isolation and analysis methodologies, the different types of biological materials (e.g., serum, plasma, urine, etc.) and the size and populations of the samples. The lack of standardization in these techniques can impact the reproducibility of results. It is also important to note that various studies quantify exosomal RNAs using different methodologies, most commonly RT-qPCR, but also next-generation sequencing (NGS) or RNA in situ hybridization (RNA-ISH). For reliable comparison of results across studies in the future, RNA levels should ideally be measured using the same technique. Moreover, an additional methodological challenge lies in the incomplete separation of exosomes from other extracellular vesicle subtypes, such as microvesicles, as well as from protein aggregates and lipoprotein particles, which often co-isolate using commonly applied techniques [110]. This overlap can affect the molecular composition detected in exosomal preparations and may partly explain the variability reported between studies, highlighting the importance of cautious interpretation of methodological differences. Furthermore, the complexity of exosomal biomarkers and their intricate networks necessitates a deeper understanding of the biological mechanisms governing their function in TNBC. Finally, the majority of studies to date concern Asian populations (primarily Chinese and Japanese), a fact that limits the generalizability of results and necessitates the performance of multi-center studies in diverse ethnic groups.

Despite these challenges, future prospects for the clinical application of exosomal biomarkers in TNBC are particularly encouraging. The next steps in research should focus on developing standardized exosome isolation and analysis protocols, as well as highly accurate and sensitive diagnostic/prognostic kits [111]. Understanding the underlying biological mechanisms through which exosomes influence TNBC development, progression, metastasis and treatment response is crucial for developing targeted interventions [112]. Additionally, research is moving towards the utilization of panels of combinatorial biomarkers over individual ones to offer greater diagnostic and prognostic accuracy [94]. Ultimately, the utilization of exosomal biomarkers will significantly contribute to achieving personalized medicine for TNBC patients, enabling early diagnosis, more accurate prognosis and more effective treatment guidance, thereby improving clinical outcomes and patient quality of life [55].

## Figures and Tables

**Table 3:** (**a**) Exosomal miRNAs and lncRNAs as predictive biomarkers for chemotherapy response in TNBC. (**b**) Exosomal proteins as predictive biomarkers for chemotherapy response in TNBC. (**c**) Exosomal PD-L1 as a predictive biomarker for immunotherapy response in TNBC.

**(a)**				
**Biomarker**	**Sample Type**	**N (Patients)/N (Controls/Others)**	**Correlation/Finding**	**Source**
SUMO1P3	serum	130 TNBC	Decreased in cases of chemosensitivity, unchanged in cases of chemoresistance in Chinese TNBC patients.	[82]
LINC00899	plasma	59 TNBC	High predictive value for pathological outcome in Chinese TNBC patients receiving neoadjuvant treatment, indicating its potential to predict chemotherapy efficacy.	[89]
circSTIL	plasma	59 TNBC	Significantly increased in Chinese TNBC patients without NAC compared to patients who received NAC.	[79]
miR-4448	serum	24 TNBC	Higher in Japanese patients with a pathological complete response to NAC.	[86]
miR-2392				
miR-2467-3p				
miR-4800-3p				
miR-127	plasma	20 BC	Upregulation associated with pCR to NAC in American BC patients.	[96]
miR-34a			After the first dose of NACT, high expression predicted non-pCR in American BC patients (6/20 TNBC).	
miR-182				
miR-183				
miR-185	plasma	34 TNBC	Lower in Spanish TNBC patients with no NACT response.	[97]
miR-4283				
miR-5008				
miR-3613				
miR-1302			Higher in Spanish TNBC patients with no NACT response.	
miR-4715				
miR-3144				
miR-770	tissue	49 TNBC	Highly expressed in chemosensitive tissues and predicted a better prognosis for Chinese TNBC.	[98]
miR-6831-5p	serum	36 TNBC	Significant difference in expression between responding and drug-resistant Chinese patients to chemotherapy.	[99]
miRNA-21	serum	53 BC	During therapy, miRNA-21 levels were directly correlated with tumor size and inversely with Ki67 expression in Spanish BC patients (13 TNBC/53).	[87]
**(b)**				
**Biomarker**	**Sample Type**	**N (Patients)/N (Controls/Others)**	**Correlation/Finding**	**Source**
ANXA6	serum	81 TNBC	Lower levels in highly sensitive Chinese TNBC patients than in resistant TNBC patients when they received first-line chemotherapy.	[100]
**(c)**				
**Biomarker**	**Sample Type**	**N (Patients)/N (Controls/Others)**	**Correlation/Finding**	**Source**
PD-L1	plasma	77 TNBC	Higher number of PD-L1 mRNA copies per mL in Italian patients with complete and partial responses compared to those with stable or progressive disease. Increase in PD-L1 mRNA copies per mL after treatment were associated with significantly shorter PFS and OS.	[101]
	plasma	902 TNBC	Predictive biomarker for ICB therapies in metastatic TNBC patients from 41 countries.	[102]

Abbreviations: BC: breast cancer, HC: healthy control, TNBC: triple-negative breast cancer, DFS: disease-free survival, OS: overall survival, NACT: neo-adjuvant chemotherapy treatment, PD-L1: programmed death-ligand 1, ICB: immune checkpoint blockade.

## Data Availability

No new data were created or analyzed in this study.
